# Palladium-Catalyzed Direct Addition of 2-Aminobenzonitriles to Sodium Arylsulfinates: Synthesis of *o*-Aminobenzophenones

**DOI:** 10.3390/molecules19056439

**Published:** 2014-05-20

**Authors:** Jiuxi Chen, Jianjun Li, Weike Su

**Affiliations:** 1 Collaborative Innovation Center of Yangtze River Delta Region Green Pharmaceuticals, College of Pharmaceutical Sciences, Zhejiang University of Technology, Hangzhou 310014, China; E-Mails: jiuxichen@163.com (C.J.); lijjun@126.com (J.L.); 2 College of Chemistry & Materials Engineering, Wenzhou University, Wenzhou 325035, China

**Keywords:** palladium, 2-aminobenzonitriles, arylsulfinates, *o*-aminobenzophenones

## Abstract

The first example of the palladium-catalyzed synthesis of *o*-aminobenzophenones in moderate to excellent yields via a direct addition of sodium arylsulfinates to unprotected 2-aminobenzonitriles was reported. A plausible mechanism for the formation of *o*-aminobenzophenones involving desulfination and addition reactions was proposed. The utility of this transformation was demonstrated by its compatibility with a wide range of functional groups. Thus, the method represents a convenient and practical strategy for synthesis of *o*-aminobenzophenones.

## 1. Introduction

*o*-Aminobenzophenones have drawn much attention due to their various pharmaceutical activities in medicine chemistry [1,2,3], their use as versatile intermediates for further transformations in synthetic chemistry [4,5,6,7,8] and their application in materials chemistry [9]. As a consequence, wide demands for diverse *o*-aminobenzophenones in various fields have promoted the development of practical and diversified synthetic methods [10,11,12,13,14,15]. Recently, Mateos reported the addition reaction of Grignard reagents to 2-aminobenzonitrile for the construction of *o*-aminobenzophenone using continuous flow chemistry [16]; however, the rigorous conditions have restricted its application and substrate diversity. Compared with Grignard reagents, sodium arylsulfinates are relatively stable, easy to handle, and are generally used as the aryl source in transition-metal-catalyzed desulfinative reactions [17,18,19,20,21]. On the other hand, transformations of nitriles play an important role in both the laboratory and industry due to their well-recognized chemical versatility [22,23]. However, the nitrile group is generally inert in organometallic reactions, and thus acetonitrile or benzonitrile usually participate as solvents or ligands [24] in metal-catalyzed reactions. The Larock group [25] pioneered the addition of arylpalladium species to the cyano group. Since then, transition metal-catalyzed addition reactions of arylation reagents to nitriles have been developed [26,27,28,29,30]. Recently, we reported the palladium-catalyzed addition of organoboron reagents to aliphatic nitriles for the preparation of alkyl aryl ketones, diketone compounds, and 2-arylbenzo[*b*]furans [31,32]. However, there is a major limitation in that trace or low yields of the desired products were observed when the substrates bore a free amino group; therefore developing a new catalyst system that would allow for the efficient reaction of problematic substrate combinations is highly desirable. These reasons may be due to side reactions and catalyst deactivation in the presence of the free amino group. In addition, nitriles bearing an electron-donating amino group, are less electrophilic, and hence addition of arylpalladium species to the cyano group ocurrs more slowly than with their electron-neutral analogues. We envisioned that electrophiles might exhibit greatly enhanced reactivity due to the formation of stable, weak coordinating and electron withdrawing cationic species by adding an appropriate additive to the reaction system.

**Scheme 1:**
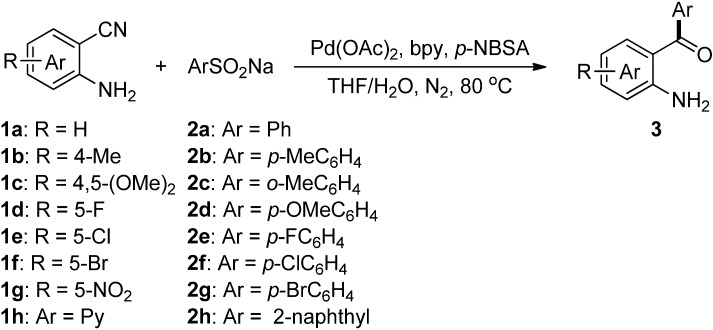
Pd-catalyzed addition of arylsulfinates to 2-aminobenzonitriles.

To the best of our knowledge, examples of *o*-aminobenzophenone synthesis using sodium arylsulfinates as coupling partners have never been reported. As part of the continuing efforts in our laboratory toward the development of palladium-catalyzed addition reactions [31,32,33,34,35,36,37], herein we report a simple and efficient protocol for the synthesis of *o*-aminobenzophenones by palladium-catalyzed direct addition of sodium arylsulfinates to unprotected 2-aminobenzonitriles (Scheme 1).

## 2. Results and Discussion

We began our study by examining the reaction between 2-aminobenzonitrile (**1a**) and sodium benzenesulfinate (**2a**) to establish the optimal reaction conditions (Table 1). On the basis of our previous addition protocol of organoborons to nitriles [31] a test reaction with Pd(O_2_CCF_3_)_2_ and 2,2'-bipyridine (bpy) as the catalytic system was performed under an air atmosphere. To our delight, the desired product *o*-aminobenzophenone (**3a**) was isolated in 18% yield (Table 1, entry 1). Encouraged by this promising result, a series of trial experiments were performed in the presence of palladium catalysts and with adjustments to the reaction parameters in order to obtain more satisfactory results. First, we investigated different palladium catalysts. Among the palladium sources used, Pd(OAc)_2_ exhibited the highest catalytic reactivity, with 32% yield (Table 1, entries 1–6). Subsequently, various additives were examined in this transformation. Screening revealed that the use of *p*-nitrobenzene-sulfonic acid (*p*-NBSA) as the additive that achieved the best result (73% yield, Table 1, entry 11). Other additives, including CF_3_CO_2_H, CH_3_CO_2_H, CH_3_SO_3_H, PhSO_3_H and *p*-toluenesulfonic acid (*p*-TSA), were less efficient (Table 1, entries 1, 7–10). We next examined the solvent effect and found that THF or 2-MeTHF were superior to dioxane, toluene, and DMF (Table 1, entries 11–15). We were pleased to discover that only when the model reaction was performed in THF under a N_2_ atmosphere did the yield dramatically increase to 91% yield (Table 1, entry 16).

**Table 1:** Optimization of the reaction conditions ^a^. 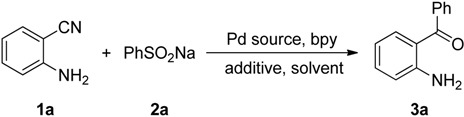

Entry	Pd source	Additive	Solvent	Yield (%) ^b^
1	Pd(CF_3_CO_2_)_2_	CF_3_CO_2_H	THF	18
2	PdCl_2_	CF_3_CO_2_H	THF	11
3	Pd(OAc)_2_	CF_3_CO_2_H	THF	32
4	Pd(acac)_2_	CF_3_CO_2_H	THF	13
5	Pd(PPh_3_)_4_	CF_3_CO_2_H	THF	trace
6	PdCl_2_(dppe)	CF_3_CO_2_H	THF	0
7	Pd(OAc)_2_	CH_3_CO_2_H	THF	10
8	Pd(OAc)_2_	CH_3_SO_3_H	THF	44
9	Pd(OAc)_2_	PhSO_3_H	THF	59
10	Pd(OAc)_2_	*p*-TSA ^c^	THF	61
11	Pd(OAc)_2_	*p*-NBSA ^d^	THF	73
12	Pd(OAc)_2_	*p*-NBSA	toluene	42
13	Pd(OAc)_2_	*p*-NBSA	2-MeTHF	69
14	Pd(OAc)_2_	*p*-NBSA	dioxane	53
15	Pd(OAc)_2_	*p*-NBSA	DMF	trace
16	Pd(OAc)_2_	*p*-NBSA	THF	91 ^e^

^a^ Reaction conditions: **1a** (0.3 mmol), **2a** (0.6 mmol), indicated Pd source (10 mol%), bpy (20 mol%), additive (3 mmol), solvent (2 mL), H_2_O (1 mL), 80 °C, 48 h, air; ^b^ Isolated yield; *^c^* *p*-TSA = *p*-toluenesulfonic acid; *^d^* *p*-NBSA = *p*-nitrobenzenesulfonic acid; ^e^ Under a N_2_ atmosphere.

With the optimized reaction conditions in hand, we next explored the substrate scope of the addition reaction of 2-aminobenzonitriles **1** with sodium arylsulfinates **2** as shown in Scheme 1.

First, the addition reaction of 2-aminobenzonitrile (**1a**) with various sodium arylsulfinates **2a**–**h** was investigated under our standard conditions (Table 2). The mono-substituent positions of the phenyl moiety of sodium arylsulfinates were evaluated, and the results demonstrated that steric effects of substituents had an obvious impact on the yield of the reaction. For example, the addition reaction of **1a **with *para*- and *ortho*-tolylsulfinate (2b and 2c) provided 87% of **3b**, while the yield of **3c**decreased to 64% (Table 2, entries 2–3). The electronic properties of the substituents on the phenyl ring of the sodium arylsulfinates also affected the yields of the reaction to some extent. In general, the sodium arylsulfinates bearing an electron-donating substituent (e.g., −Me and −OMe) produced slightly higher yields than those analogues bearing an electron-withdrawing substituent (e.g., −F, −Cl and −Br) (Table 2, entries 2, 4–7). Substrate **2h**, bearing a naphthyl group, was treated with **1a** to deliver the desired product **3h** in 90% yield (Table 2, entry 8). It is noteworthy that the fluoro, chloro, and bromo moieties (commonly used for cross-coupling reactions) in substrates were all tolerated and afforded several halogen-containing products **3e**–**g** (Table 2, entries 5–7) in acceptable yields, leading to a useful handle for further cross-coupling reactions. However, treatment of an alkylsulfinate such as sodium methanesulfinate with **1a** under the optimized conditions afforded only a trace amount of the desired product.

**Table 2:** Substrate scope of sodium arylsulfinates ^a^. 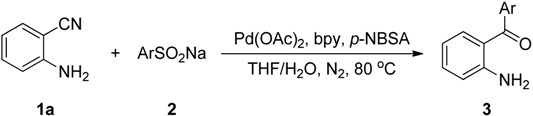

Entry	ArSO_2_Na (2)	Product (3)	Yield (%) ^b^
1	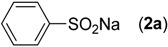	**3a**	91
2	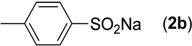	**3b**	88
3	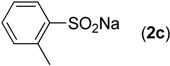	**3c**	64
4	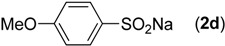	**3d**	85
5	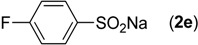	**3e**	81
6	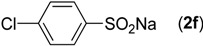	**3f**	83
7	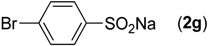	**3g**	80
8	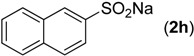	**3h**	90

^a^ *Reaction conditions:* **1a** (0.3 mmol), **2** (0.6 mmol), Pd(OAc)_2_ (10 mol%), bpy (20 mol%), *p*-NBSA (3 mmol), THF (2 mL), H_2_O (1 mL), 80 °C, 48 h, N_2_; ^b^ Isolated yield.

**Scheme 2:**
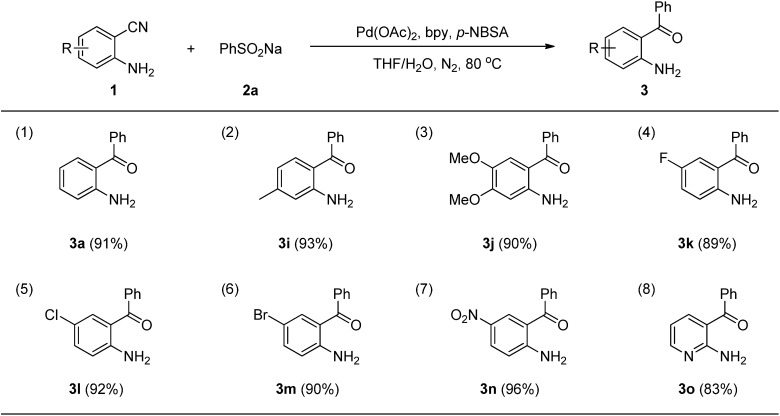
Substrate scope of 2-aminobenzonitriles ^a^.

Next, we turned our attention to the effect of the reactions of sodium benzenesulfinate (**2a**) with various 2-aminobenzonitriles (**1a**–**h**) under our standard conditions and the results are summarized in Scheme 2. As expected, the groups on the phenyl ring of 2-aminobenzonitriles, such as methyl, methoxy, fluoro, chloro, bromo, and nitro were quite compatible. The electronic properties of the groups on the phenyl ring moiety of 2-aminobenzonitriles had little effect on the reaction. For example, substrates **1b** and **1c** bearing an electron-donating substituent (e.g., −Me or −OMe), reacted with **2a** smoothly and afforded the corresponding products **3i** and **3j** in 93% and 90% yields, respectively (Scheme 2, entries 2–3). Substrates **1d**, **1e**, **1f** and **1g** bearing an electron-withdrawing substituent (e.g., −F, −Cl, −Br and −NO_2_) were treated with **2a** to afford 89%, 92%, 90% and 96% yields of **3k**, **3l**, **3m** and **3n**, respectively (Scheme 2, entries 4–7). Gratifyingly, the substrate 2-aminonicotinonitrile (**1h**), bearing a heteroaryl group underwent the reaction smoothly to afford the corresponding product **3o** in 83% yield (Scheme 2, entry 8).

*^a^* *Reaction conditions*: **1** (0.3 mmol), **2a** (0.6 mmol), Pd(OAc)_2_ (10 mol%), bpy (20 mol%), *p*-NBSA (3 mmol), THF (2 mL), H_2_O (1 mL), 80 °C, 48 h, N_2_; Isolated yield was given in parenthesis.

**Scheme 3:**
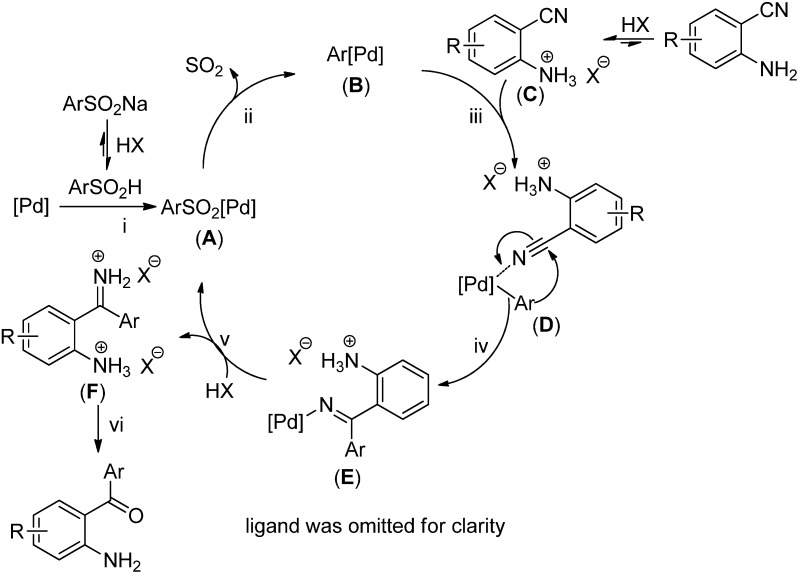
Proposed mechanism.

A plausible mechanism for the formation of *o*-aminobenzophenones is proposed in Scheme 3. The following key steps are included in the catalytic pathway: (i) coordination of Pd(OAc)_2_ with arylsulfinic acids (or sodium arylsulfinates) to afford a palladium species **A**; (ii) the desulfination of the arylsulfinic acid to give aryl-palladium species **B**; (iii) the formation of intermediate **D** by the coordination of species **B** with cyano group in 2-cyanobenzenaminium (**C**); (iv) carbopalladation of the 2-aminobenzonitriles to produce the corresponding ketimine complex **E**; (v) protonation of the ketimine complex **E** to afford the ketimine intermediate **F** and regenerate an active palladium species. Hydrolysis of the ketimine intermediate **F** delivers the corresponding *o*-aminobenzophenones as the desired products.

## 3. Experimental

### General Information

Melting points are uncorrected. ^1^H-NMR and ^13^C-NMR spectra were measured on a 500 MHz spectrometer using CDCl_3_ as the solvent with tetramethylsilane (TMS) as an internal standard at room temperature. Chemical shifts are given n *δ* relative to TMS, and the coupling constants *J* are given in hertz. Other commercially obtained reagents were used without further purification. All reactions under N_2_atmosphere were conducted using standard Schlenk techniques. Column chromatography was performed using EM silica gel 60 (300–400 mesh).

### General Procedure for the Synthesis of o-Aminobenzophenones

Under a N_2_ atmosphere, a Schlenk tube was charged with 2-aminobenzonitrile **1** (0.3 mmol), sodium arylsulfinate **2** (0.6 mmol), Pd(OAc)_2_ (10 mol %), bpy (20 mol %), *p*-NBSA (10 equiv), THF (2 mL), and H_2_O (1 mL) at room temperature. The reaction mixture was stirred vigorously at 80 °C for 48 h. The mixture was poured into ethyl acetate, which was washed with saturated NaHCO_3_ (2 × 10 mL) and then brine (1 × 10 mL). After the aqueous layer was extracted with ethyl acetate, the combined organic layers were dried over anhydrous MgSO_4_ and evaporated under reduced pressure. The residue was purified by flash column chromatography (hexane/ethyl acetate) to afford the desired products **3**.

*2-Aminobenzophenone* (**3a**). Pale yellow solid (91% yield), mp 110–112 °C (Lit. [13] 109–111 °C); ^1^H-NMR (CDCl_3_, 500 MHz): δ 7.63–7.64 (m, 2H), 7.31–7.54 (m, 4H), 7.26–7.28 (m, 1H), 6.74 (d, *J* = 8.3 Hz, 1H), 6.60 (t, *J* = 7.6 Hz, 1H), 6.09 (s, 2H); ^13^C-NMR (CDCl_3_, 125 MHz) δ 199.1, 150.9, 140.1, 134.6, 134.2, 131.0, 129.1, 128.1, 118.2, 117.0, 115.5.

*(2-Aminophenyl)(p-tolyl)methanone* (**3b**) [5]. Pale yellow solid (88% yield), mp 92–93 °C (not reported); ^1^H-NMR (CDCl_3_, 500 MHz): δ 7.56 (d, *J* = 8.1 Hz, 2H), 7.45 (d, *J* = 8.0 Hz, 1H), 7.25–7.30 (m, 3H), 6.73 (d, *J* = 8.2 Hz, 1H), 6.60 (t, *J* = 7.5 Hz, 1H), 6.00 (s, 2H), 2.42 (s, 3H); ^13^C-NMR (CDCl_3_, 125 MHz) δ 198.8, 150.7, 141.7, 137.2, 134.4, 134.0, 129.4, 128.7, 118.6, 116.9, 115.5, 21.5.

*(2-Aminophenyl)(o-tolyl)methanone* (**3c**). Pale yellow solid (64% yield), mp 79–81 °C (Lit. [38] 84 °C); ^1^H-NMR (CDCl_3_, 500 MHz): δ 7.32–7.35 (m, 1H), 7.20–7.29 (m, 5H), 6.71 (d, *J* = 8.3 Hz, 1H), 6.52 (t, *J* = 7.6 Hz, 1H), 6.41 (s, 2H), 2.27 (s, 3H); ^13^C-NMR (CDCl_3_, 125 MHz) δ 200.8, 150.7, 140.1, 134.6, 134.3, 134.2, 130.0, 128.7, 126.6, 124.7, 117.9, 116.4, 115.1, 19.0.

*(2-Aminophenyl)(4-methoxyphenyl)methanone* (**3d**). Pale yellow solid (85% yield), mp 77–78 °C (Lit. [4] 75–76 °C); ^1^H-NMR (CDCl_3_, 500 MHz ): δ 7.68 (d, *J* = 8.8 Hz, 2H), 7.46 (d, *J* = 8.0 Hz, 1H), 7.28 (t, *J* = 7.7 Hz, 1H), 6.95 (d, *J* = 8.8 Hz, 2H), 6.73 (d, *J* = 8.3 Hz, 1H), 6.62 (t, *J* = 7.1 Hz, 1H), 5.86 (s, 2H), 3.38 (s, 3H); ^13^C-NMR (CDCl_3_, 125 MHz) δ 197.8, 162.3, 150.4, 134.0, 133.7, 131.8, 122.2, 199.0, 117.0, 115.6, 113.4, 55.4.

*(2-Aminophenyl)(4-fluorophenyl)methanone* (**3e**) [6]. Pale yellow solid (81% yield), mp 128–129 °C (not reported); ^1^H-NMR (CDCl_3_, 500 MHz): δ 7.67–7.69 (m, 2H), 7.42 (d, *J* = 8.1 Hz, 1H), 7.29–7.32 (m, 1H), 7.14 (t, *J* = 8.7 Hz, 2H), 6.75 (d, *J* = 8.3 Hz, 1H), 6.62 (t, *J* = 7.6 Hz, 1H), 6.03 (s, 2H); ^13^C-NMR (CDCl_3_, 125 MHz) δ 197.5, 165.5, 163.5, 150.8, 136.1, 134.3, 134.2, 131.7, 131.6, 118.1, 117.1, 115.6, 115.3, 115.1.

*(2-Aminophenyl)(4-chlorophenyl)methanone* (**3f**) [5]. Pale yellow solid (83% yield), mp 100–101 °C (not reported); ^1^H-NMR (CDCl_3_, 500 MHz): δ 7.59 (d, *J* = 8.5 Hz, 2H), 7.31–7.44 (m, 3H), 7.26–7.28 (m, 1H), 6.73 (d, *J* = 8.9 Hz, 1H), 6.60 (t, *J* = 7.6 Hz, 1H), 6.08 (s, 2H); ^13^C-NMR (CDCl_3_, 125 MHz) δ 197.9, 151.0, 138.4, 137.4, 134.5, 134.3, 130.6, 128.4, 117.9, 117.2, 115.7.

*(2-Aminophenyl)(4-bromophenyl)methanone* (**3g**) [5]. Pale yellow solid (80% yield), mp 109–111 °C (not reported); ^1^H-NMR (CDCl_3_, 500 MHz): δ 7.60 (d, *J* = 8.5 Hz, 2H), 7.51 (d, *J* = 8.5 Hz, 2H), 7.40 (d, *J* = 8.1 Hz, 1H), 7.27–7.32 (m, 1H), 6.74 (d, *J* = 8.3 Hz, 1H), 6.61 (t, *J* = 7.6 Hz, 1H), 6.10 (s, 2H); ^13^C-NMR (CDCl_3_, 125 MHz) δ 197.8, 151.0, 138.8, 134.5, 134.2, 131.4, 130.7, 125.8, 117.7, 117.1, 115.6.

*(2-Aminophenyl)(naphthalen-2-yl)methanone* (**3h**). Pale yellow solid (90% yield), mp 107–108 °C (Lit. [38] 106 °C); ^1^H-NMR (CDCl_3_, 500 MHz ): δ 8.12 (s, 1H), 7.89–7.93 (m, 3H), 7.77 (d, *J* = 8.5 Hz, 1H), 7.51–7.60 (m, 3H), 7.30–7.31 (m, 1H), 6.77 (d, *J* = 8.3 Hz, 1H), 6.62 (t, *J* = 7.1 Hz, 1H), 6.09 (s, 2H); ^13^C-NMR (CDCl_3_, 125 MHz) δ 199.0, 150.9, 137.3, 134.6, 134.6, 134.2, 132.3, 130.1, 129.1, 128.0, 127.8, 127.7, 126.7, 125.8, 118.5, 117.1, 115.6.

*(2-Amino-4-methylphenyl)(phenyl)methanone* (**3i**) [5]. Pale yellow solid (93% yield), mp 67–68 °C (not reported); ^1^H-NMR (CDCl_3_, 500 MHz ): δ 7.61 (d, *J* = 8.4 Hz, 2H), 7.44–7.52 (m, 3H), 7.33 (d, *J* = 8.2 Hz, 1H), 6.54 (s, 1H), 6.41 (d, *J* = 7.3 Hz, 1H), 6.12 (s, 2H), 2.29 (s, 3H); ^13^C-NMR (CDCl_3_, 125 MHz) δ 198.7, 151.3, 145.4, 140.5, 134.8, 130.8, 129.0, 128.1, 117.1, 117.0, 116.0, 21.8.

*(2-Amino-4,5-dimethoxyphenyl)(phenyl)methanone* (**3j**) [7]. Pale yellow solid (90% yield), mp 79–81 °C (not reported); ^1^H-NMR (CDCl_3_, 500 MHz): δ 7.61 (d, *J* = 8.4 Hz, 2H), 7.43–7.51 (m, 3H), 6.92 (s, 1H), 6.25 (s, 2H), 6.20 (s, 1H), 3.88 (s, 3H), 3.65 (s, 3H); ^13^C-NMR (CDCl_3_, 125 MHz) δ 197.2, 155.5, 148.6, 140.7, 139.7, 130.6, 128.7, 128.1, 116.7, 110.0, 99.3, 56.6, 55.9.

*(2-Amino-5-fluorophenyl)(phenyl)methanone* (**3k**) [8]. Pale yellow solid (89% yield), mp 117–118 °C (not reported); ^1^H-NMR (CDCl_3_, 500 MHz): δ 7.46–7.65 (m, 5H), 7.05–7.16 (m, 2H), 6.70–6.72 (m, 1H), 5.91 (s, 2H); ^13^C-NMR (CDCl_3_, 125 MHz) δ 198.0, 154.1, 152.2, 147.3, 139.4, 131.5, 129.1, 128.3, 122.3, 122.1, 119.1, 118.9, 118.2, 118.1, 117.9.

*(2-Amino-5-chlorophenyl)(phenyl)methanone* (**3l**) [5]. Pale yellow solid (92% yield), mp 97–98 °C (not reported); ^1^H-NMR (CDCl_3_, 500 MHz ): δ 7.63 (d, *J* = 8.4 Hz, 2H), 7.47–7.56 (m, 3H), 7.41 (d, *J* = 2.5 Hz, 1H), 7.23–7.25 (m, 1H), 6.69 (d, *J* = 8.8 Hz, 1H), 6.07 (s, 2H); ^13^C-NMR (CDCl_3_, 125 MHz) δ 198.0, 149.4, 139.3, 134.2, 133.3, 131.6, 129.1, 128.4, 120.0, 118.8, 118.5.

*(2-Amino-5-bromophenyl)(phenyl)methanone* (**3m**) [7]. Pale yellow solid (90% yield), mp 109–110 °C (not reported); ^1^H-NMR (CDCl_3_, 500 MHz): δ 7.63 (d, *J* = 8.4 Hz, 2H), 7.47–7.58 (m, 4H), 7.35–7.37 (m, 1H), 6.65 (d, *J* = 8.8 Hz, 1H), 6.10 (s, 2H); ^13^C-NMR (CDCl_3_, 125 MHz) δ 198.0, 149.8, 139.4, 136.9, 136.3, 131.7, 129.2, 128.5, 119.6, 118.9, 106.7.

*(2-Amino-5-nitrophenyl)(phenyl)methanone* (**3n**) [5]. Pale yellow solid (96% yield), mp 151–152 °C (not reported); ^1^H-NMR (CDCl_3_, 500 MHz ): δ 8.48 (s, 1H), 8.17 (d, *J* = 9.2 Hz, 1H), 7.51–7.66 (m, 5H), 6.90 (s, 2H), 6.76 (d, *J* = 9.2 Hz, 1H); ^13^C-NMR (CDCl_3_, 125 MHz) δ 198.0, 155.3, 138.5, 136.7, 132.2, 131.6, 129.3, 129.2, 128.7, 116.8, 116.1.

*(2-Aminopyridin-3-yl)(phenyl)methanone* (**3o**). Pale yellow solid (83% yield), mp 143–144 °C (Lit. [39] 140 °C); ^1^H-NMR (CDCl_3_, 500 MHz): δ 8.25 (d, *J* = 4.8 Hz, 2H), 7.76–7.77 (m, 1H), 7.48–7.62 (m, 5H), 6.82 (s, 2H), 6.59–6.62 (m, 1H); ^13^C-NMR (CDCl_3_, 125 MHz) δ 197.8, 159.8, 153.9, 143.0, 139.2, 131.6, 129.1, 128.4, 112.9, 112.1.

## 4. Conclusions

In summary, we have developed a new strategy for constructing *o*-aminobenzophenones in moderate to excellent yields via palladium-catalyzed direct addition reaction of sodium arylsulfinates to unprotected 2-aminobenzonitriles. Further efforts to extend this catalytic system to the preparation of other useful compounds are currently underway in our laboratories.

## Supplementary Materials

Supplementary materials can be accessed at: http://www.mdpi.com/1420-3049/19/5/6439/s1.
