# Supplementary figures and images for: Palladium-Catalyzed Direct Addition of 2-Aminobenzonitriles to Sodium Arylsulfinates: Synthesis of o-Aminobenzophenones
Source: Molecules. 2014 May 20;19(5):6439–49. doi: 10.3390/molecules19056439 (PMC6271184; doi:10.3390/molecules19056439)

# Supplementary File

Copies of  $^1\text{H}$ -NMR and  $^{13}\text{C}$ -NMR for all products.

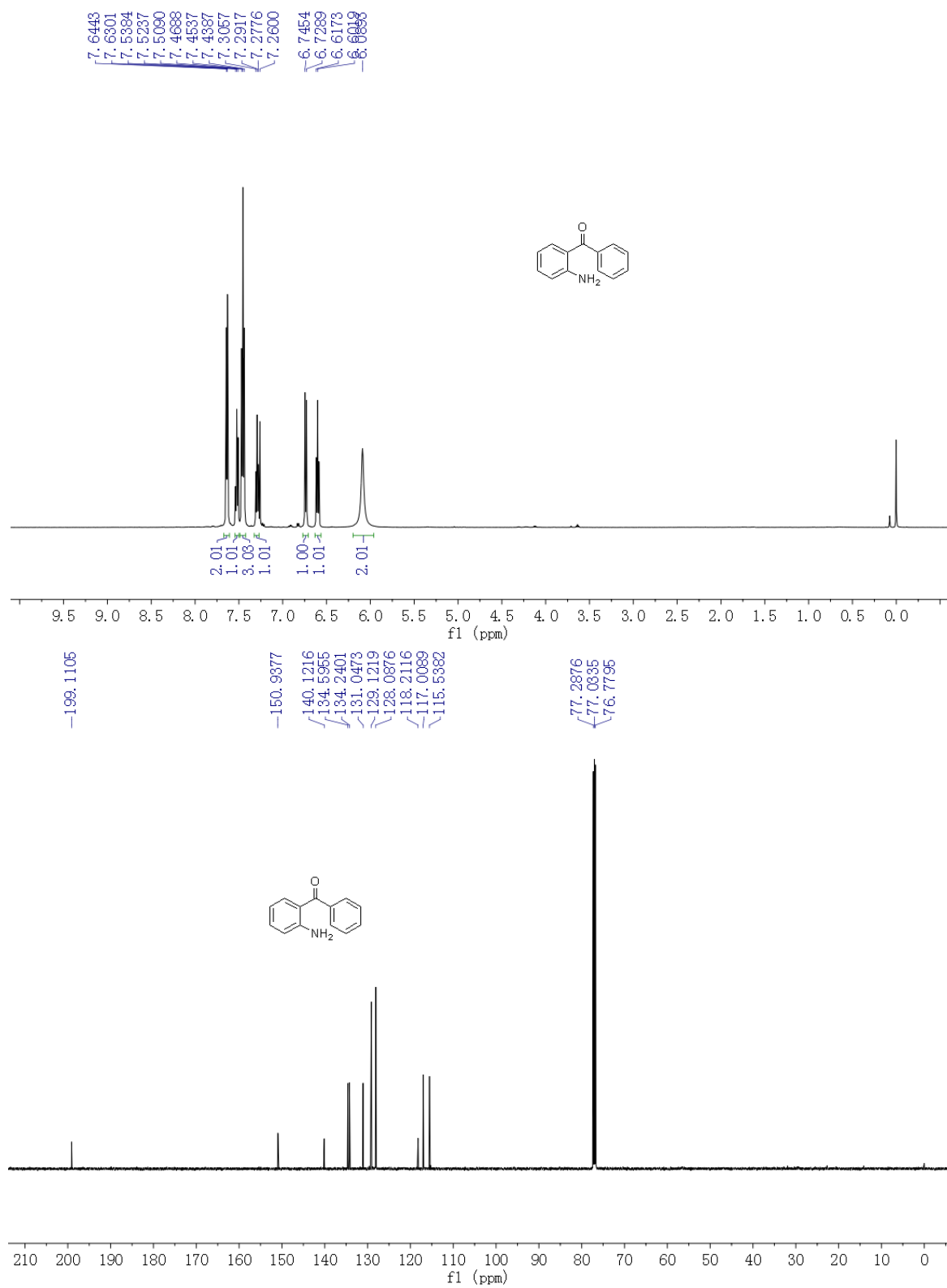

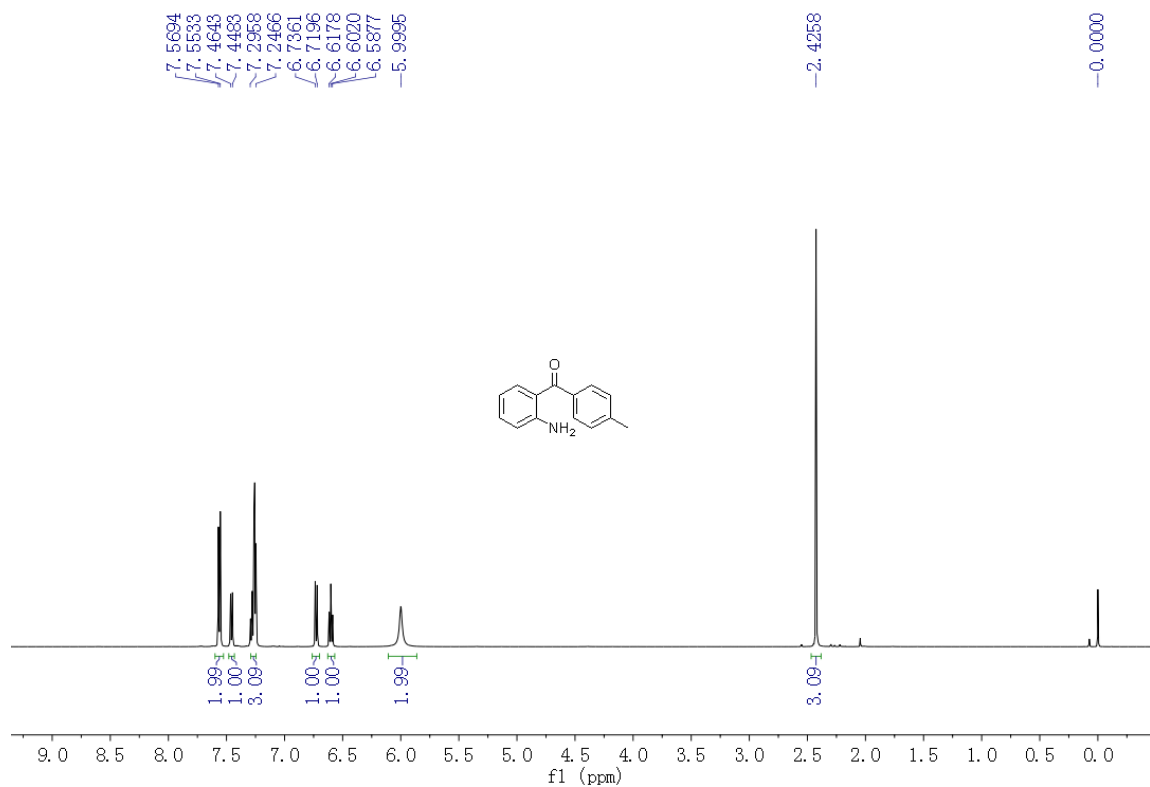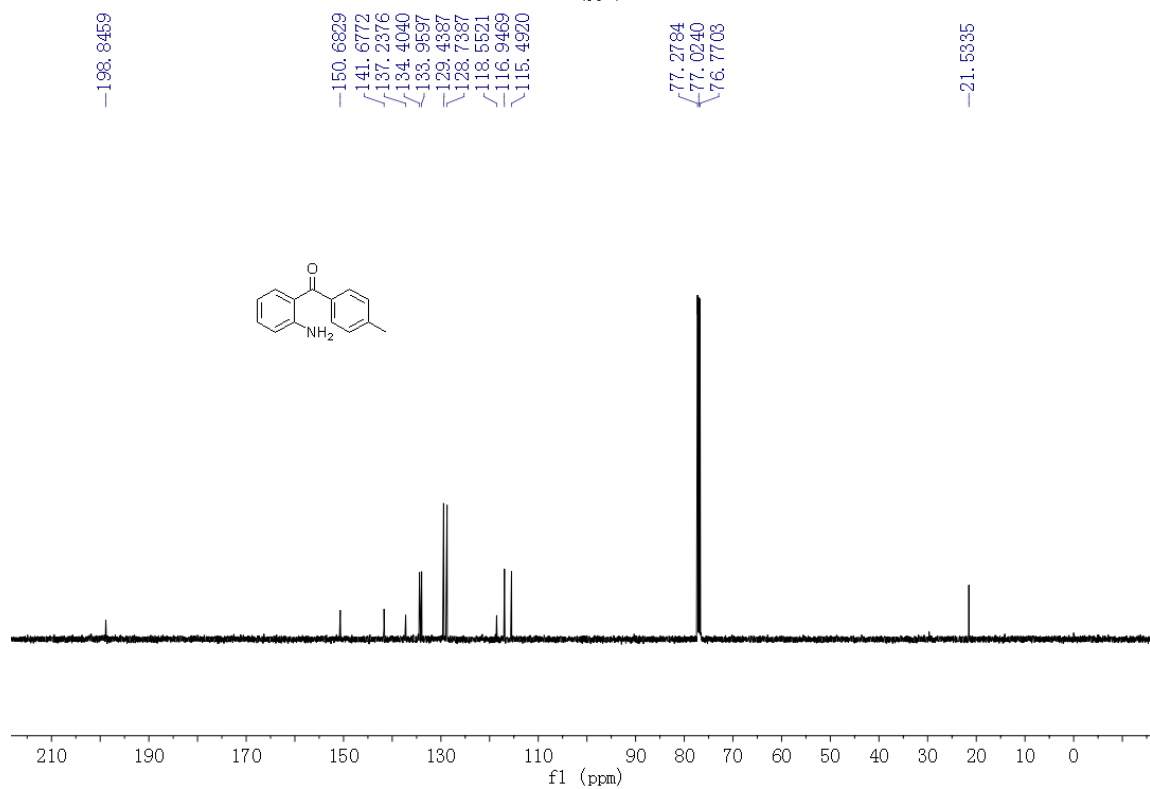

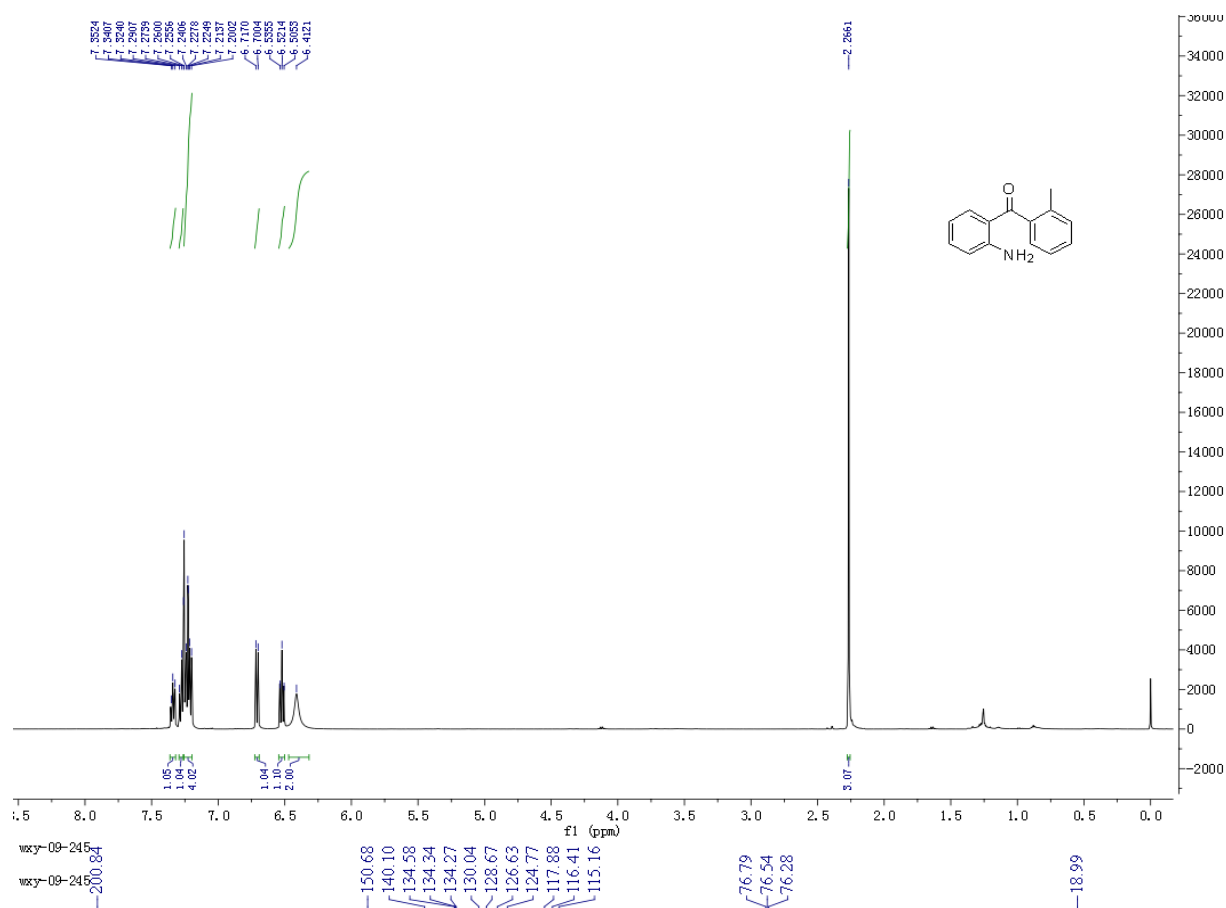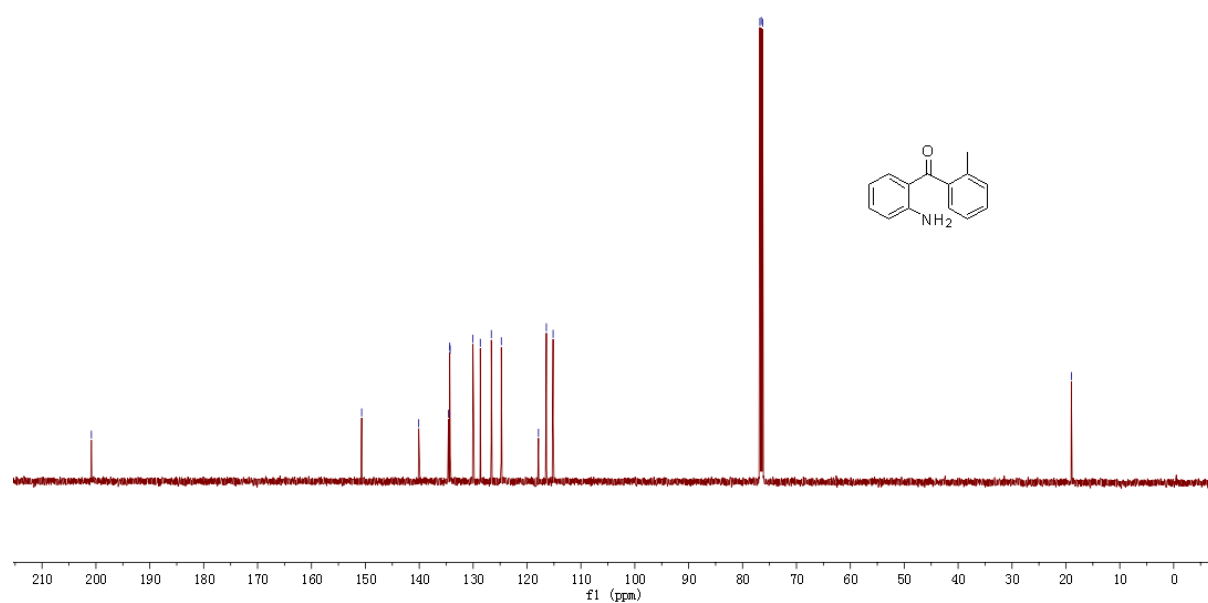

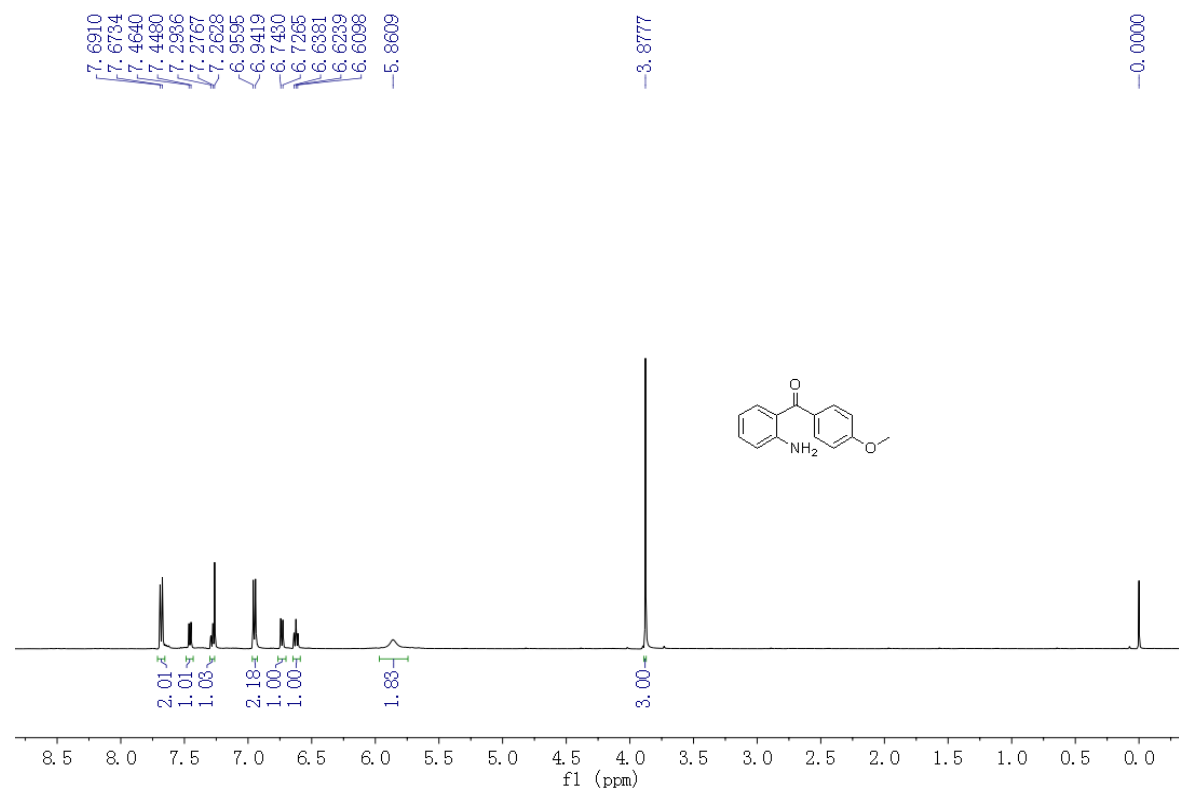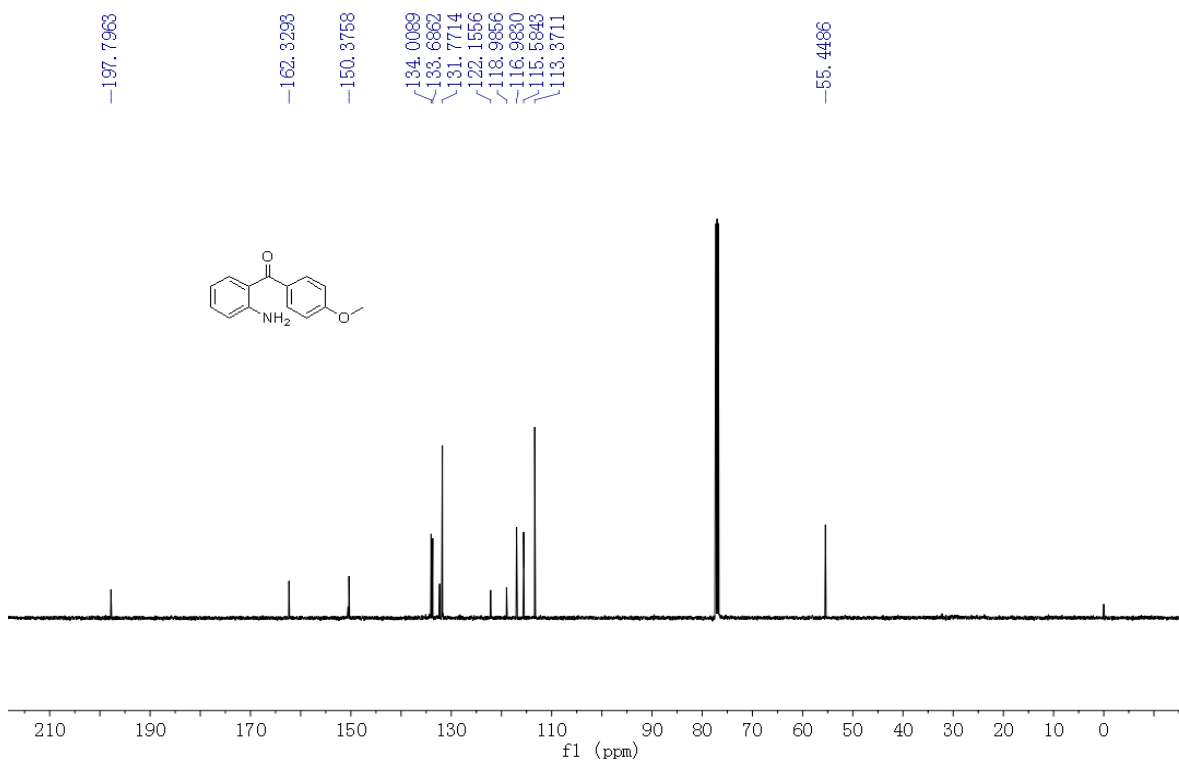

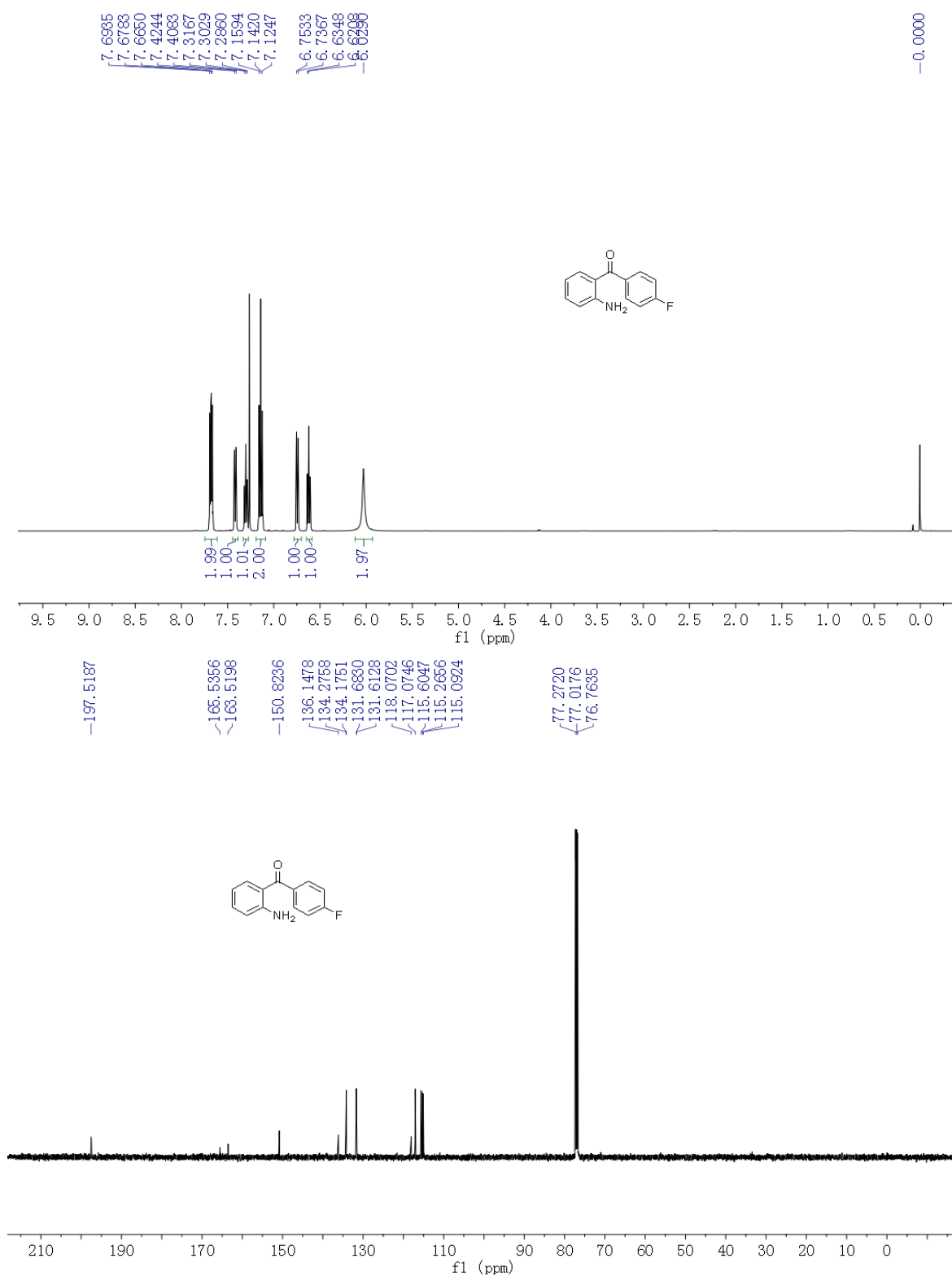

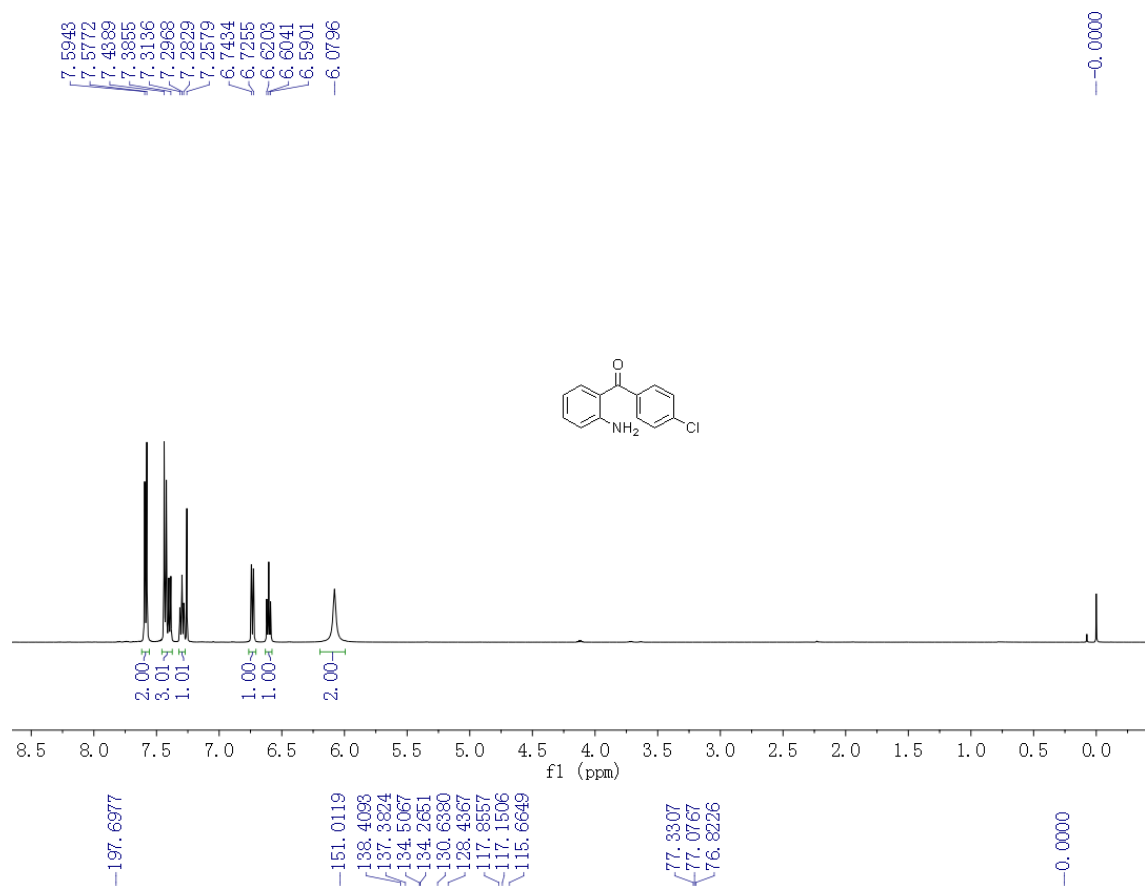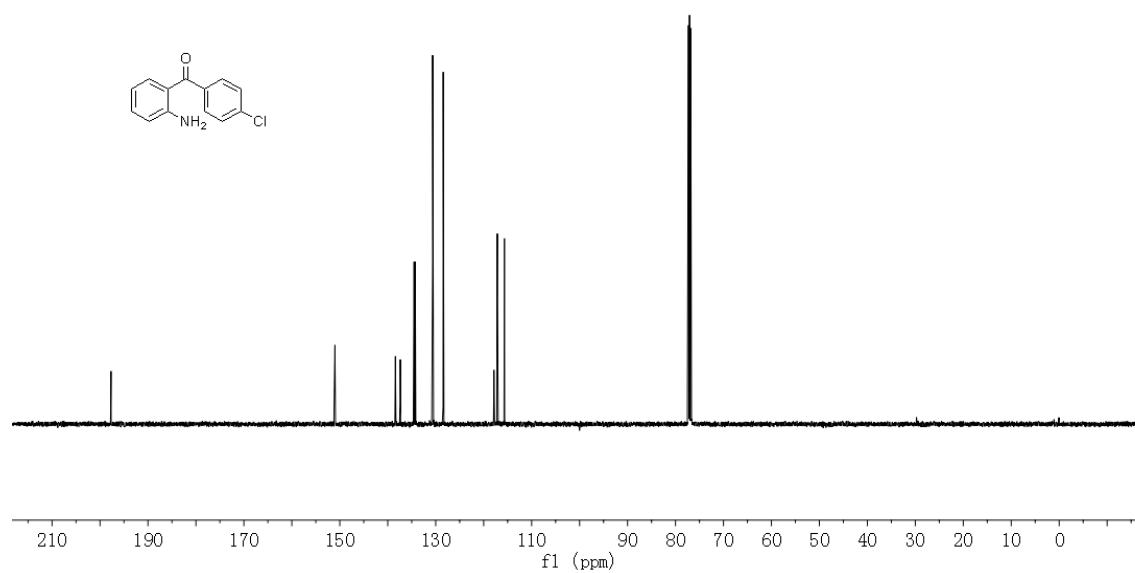

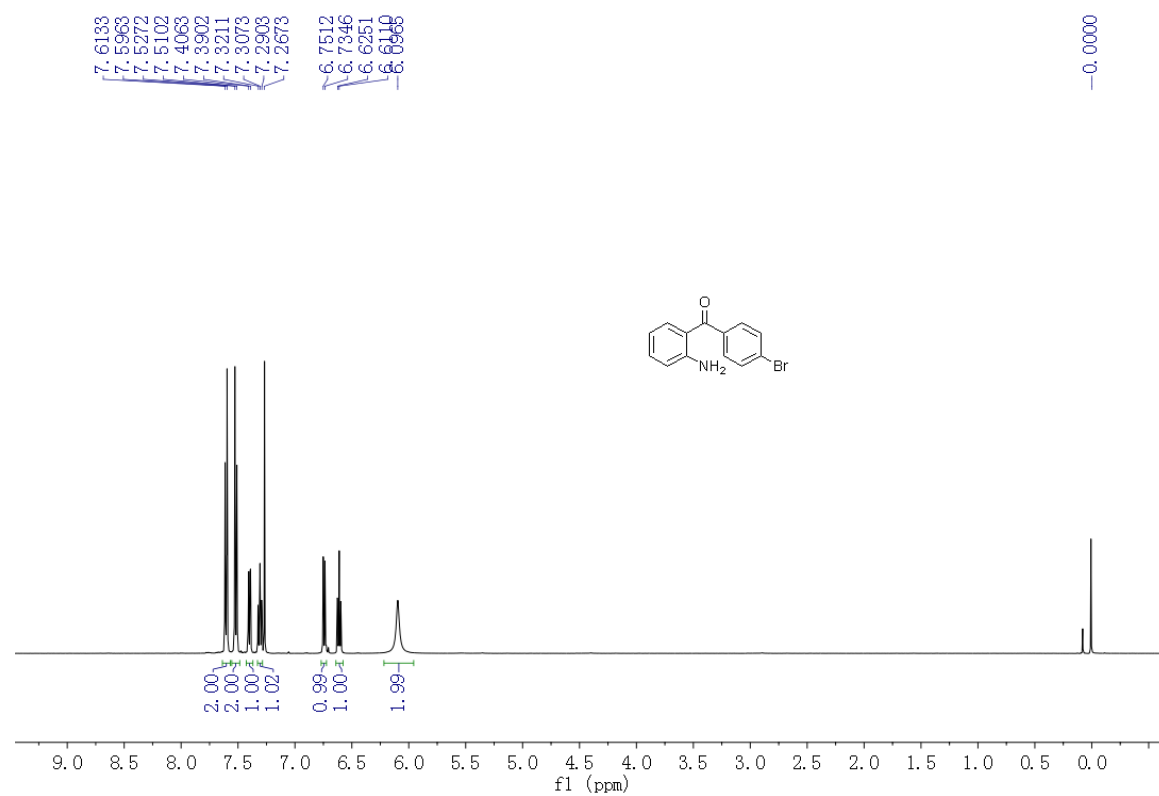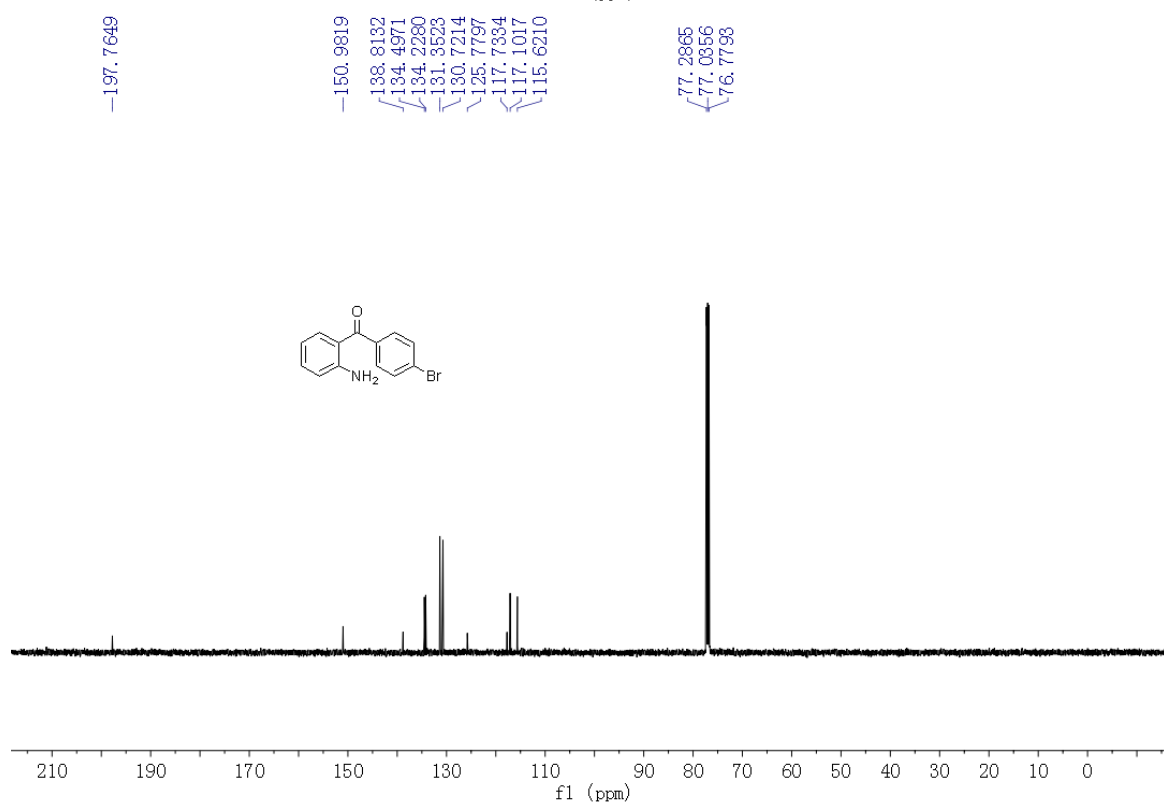

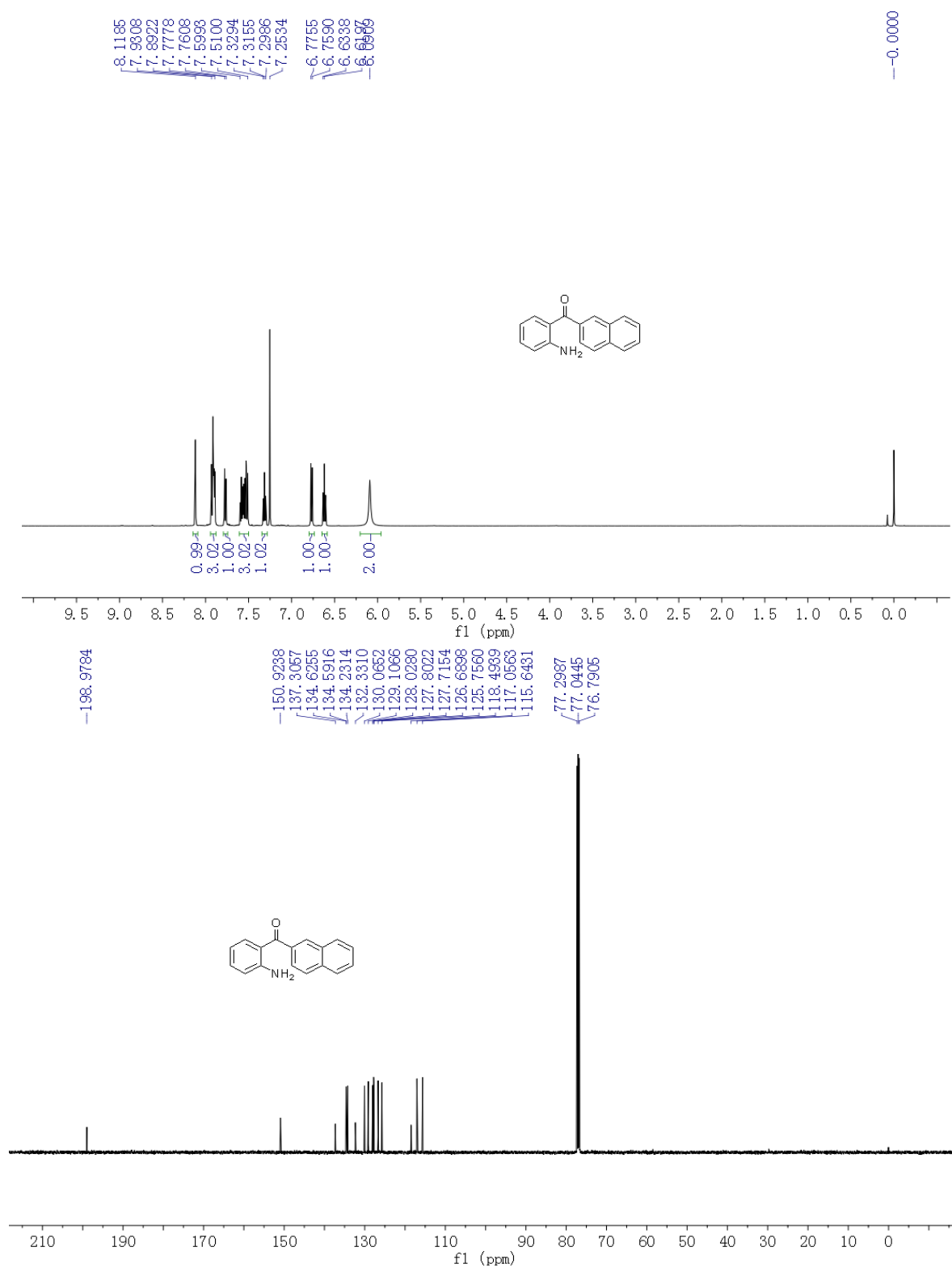

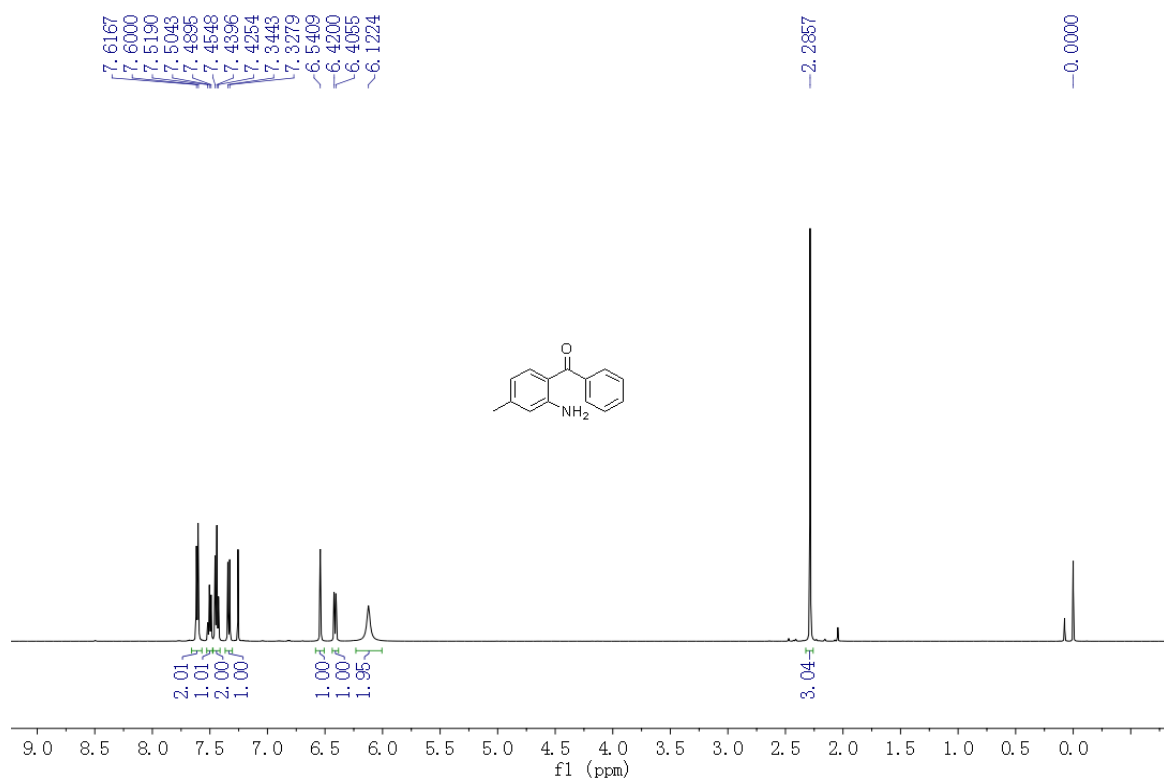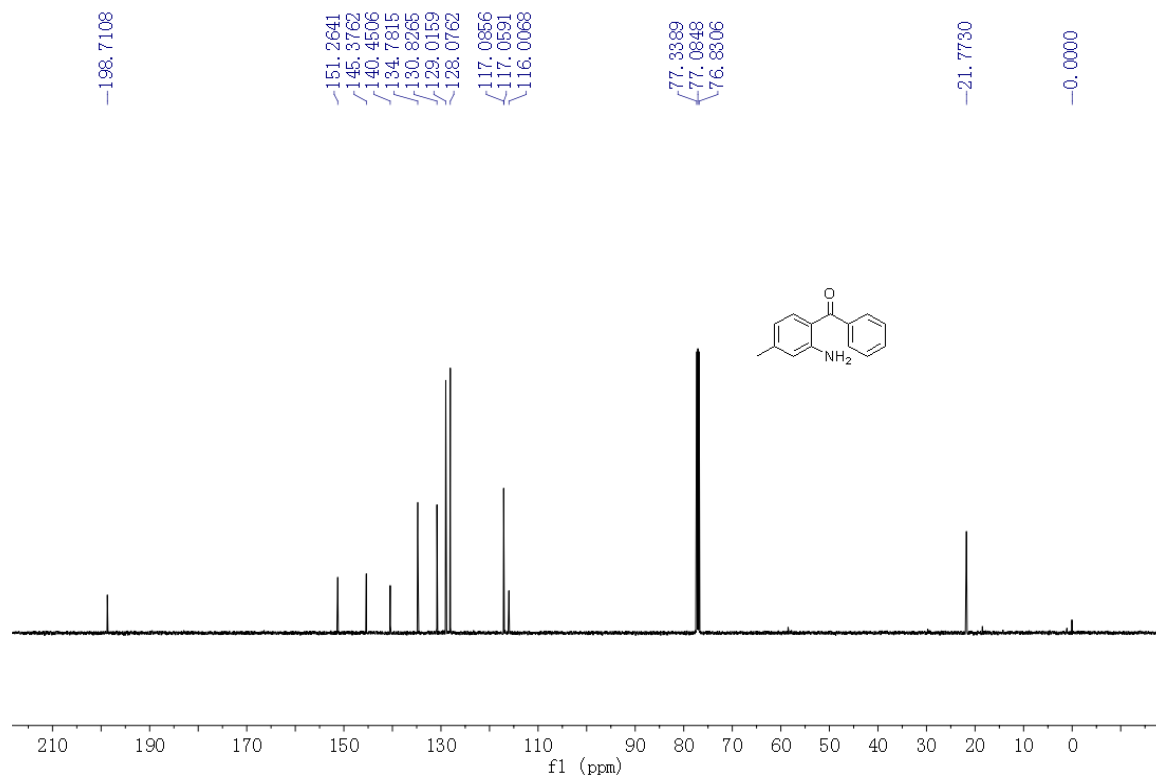

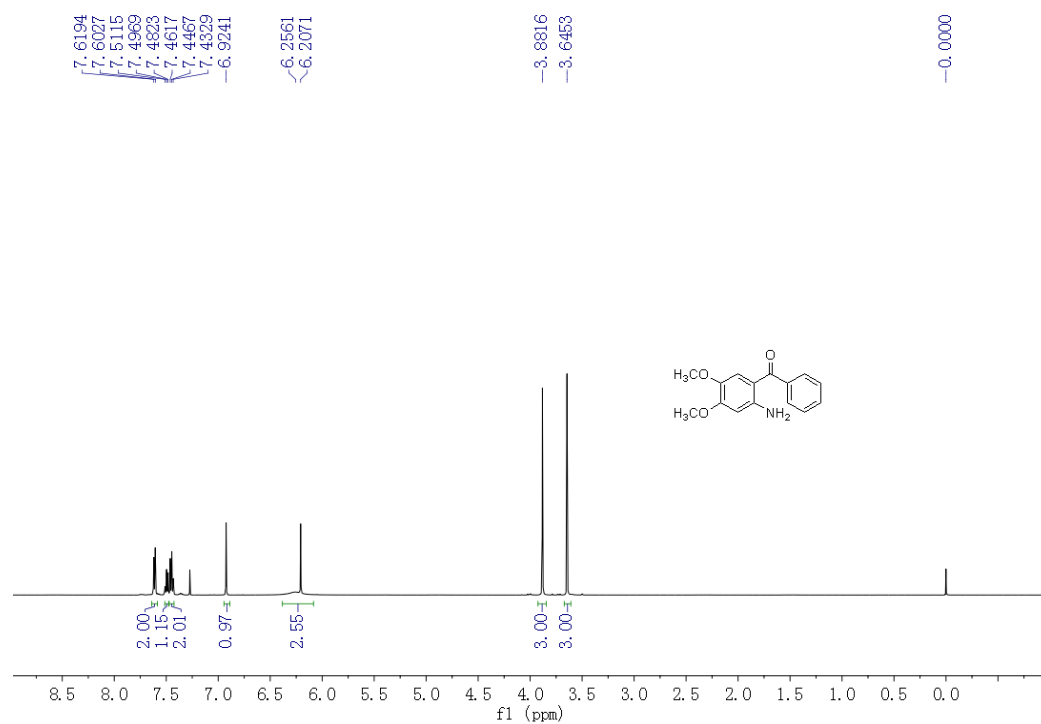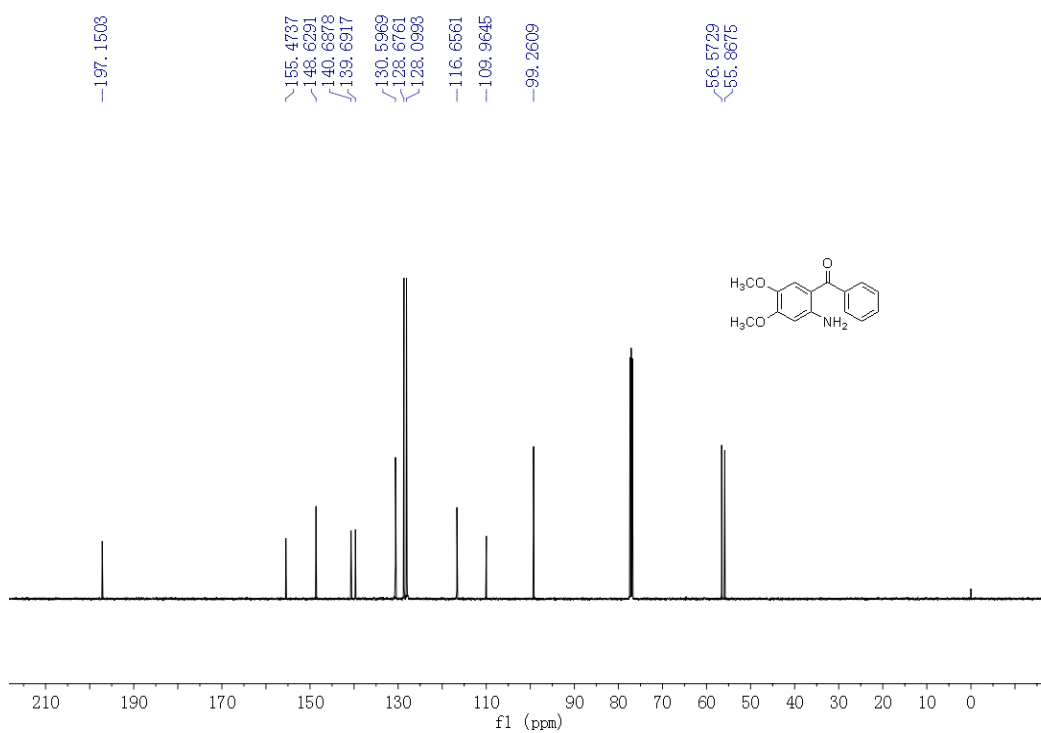

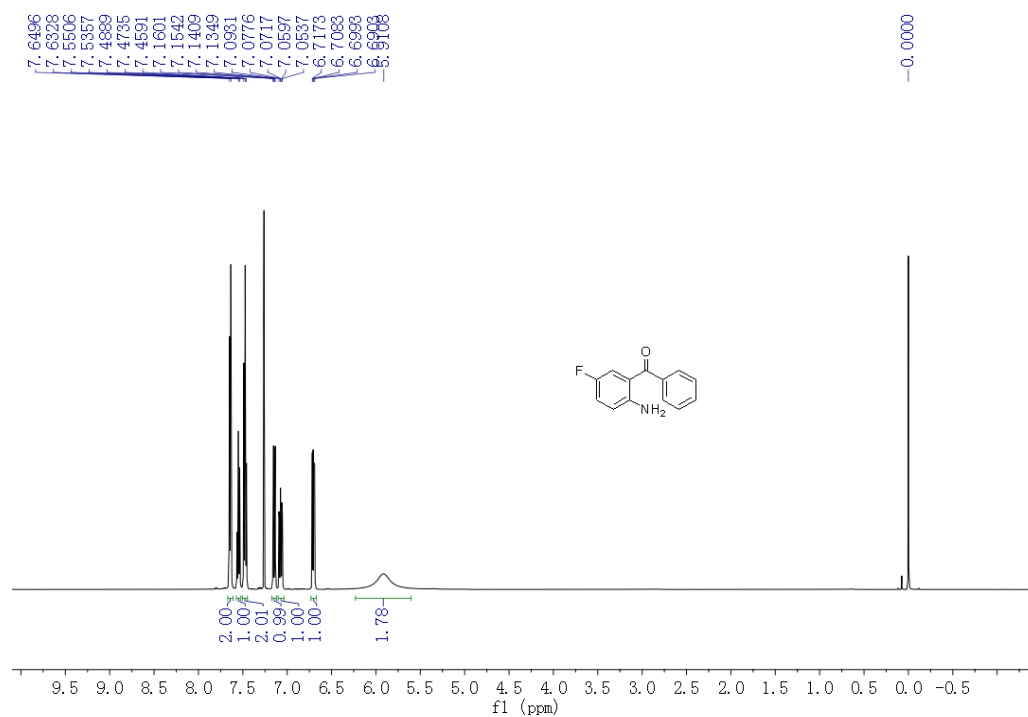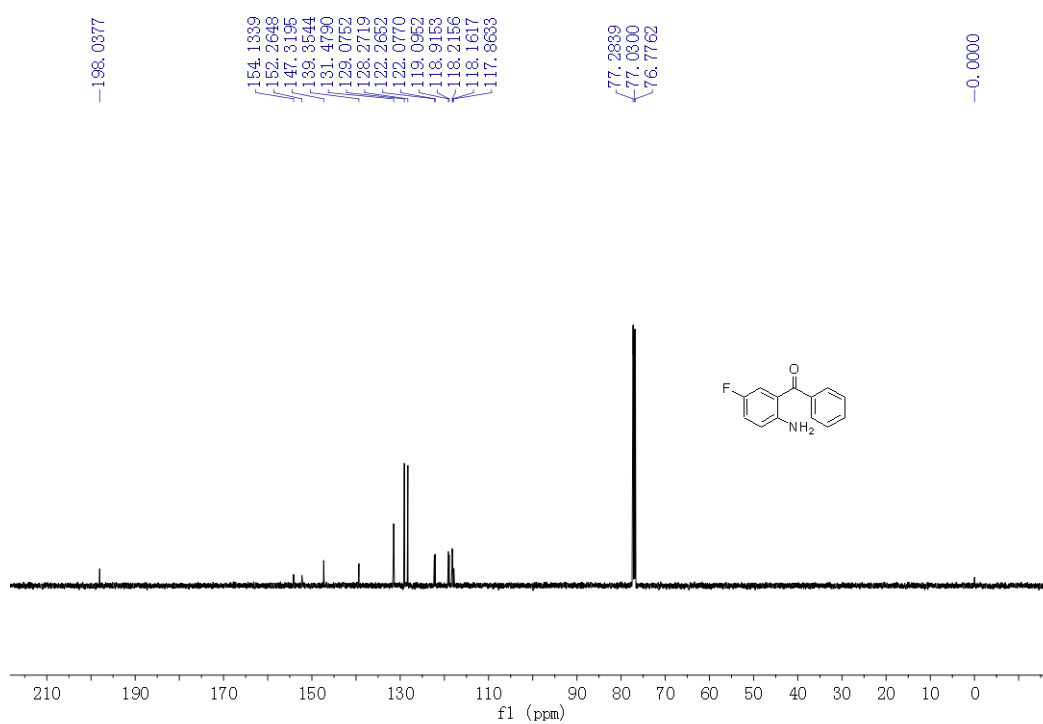

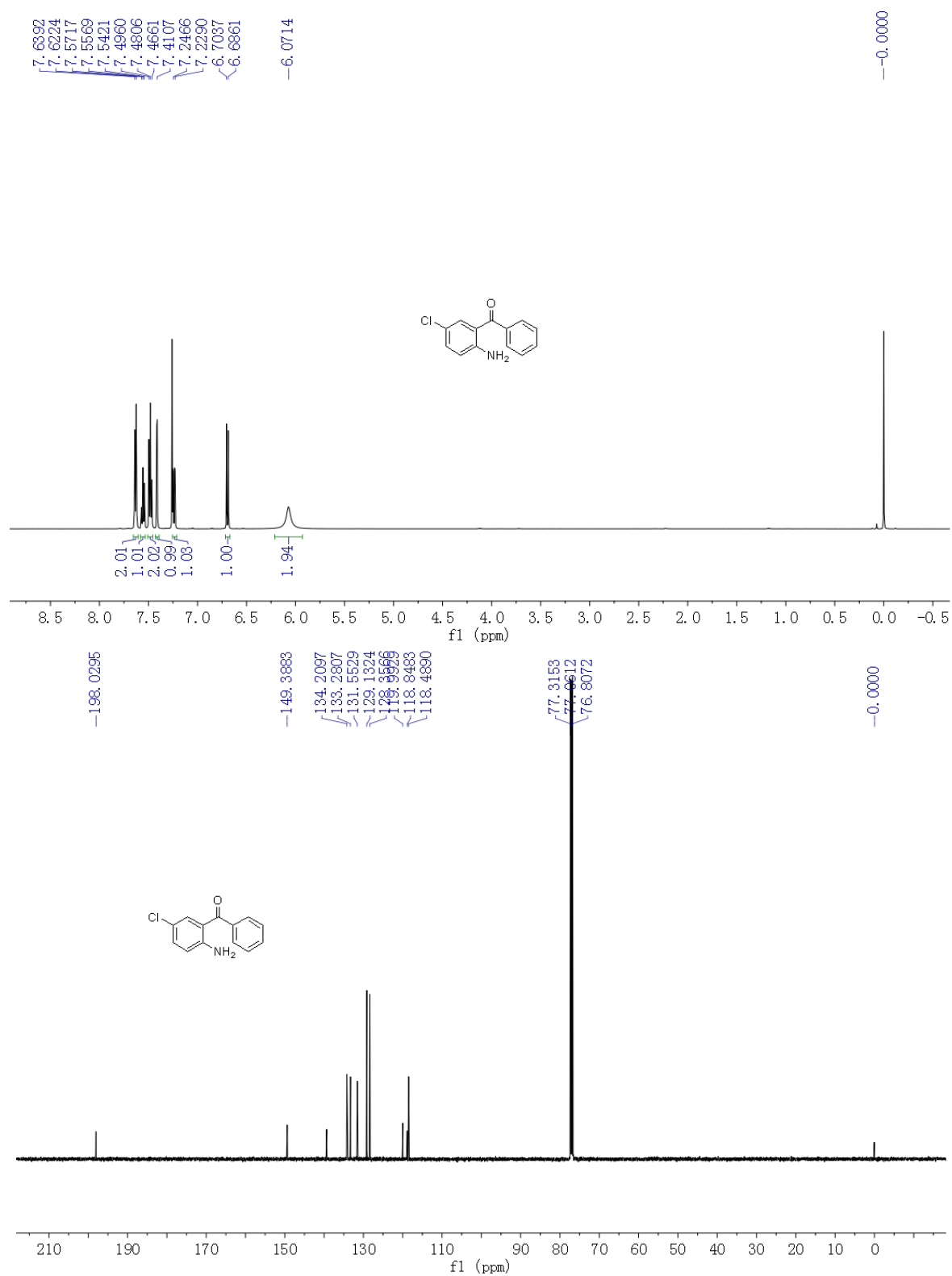

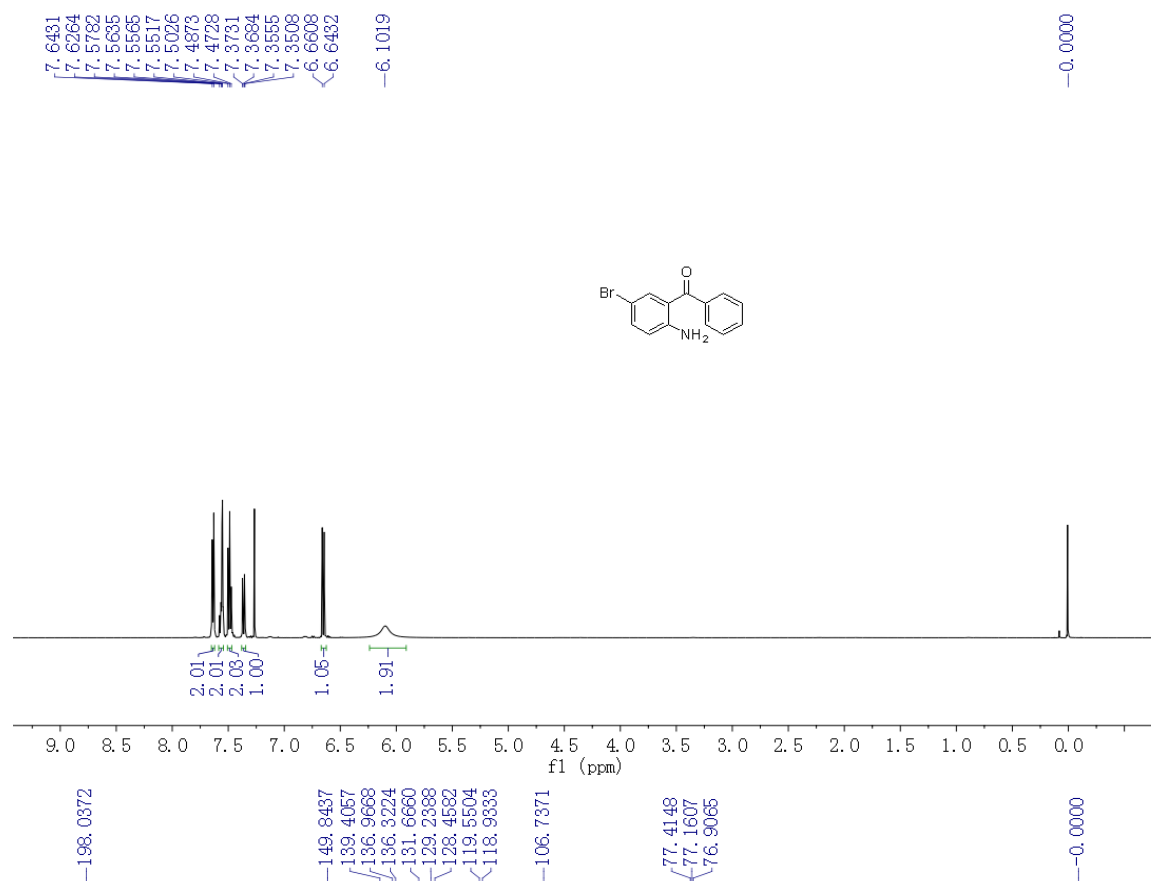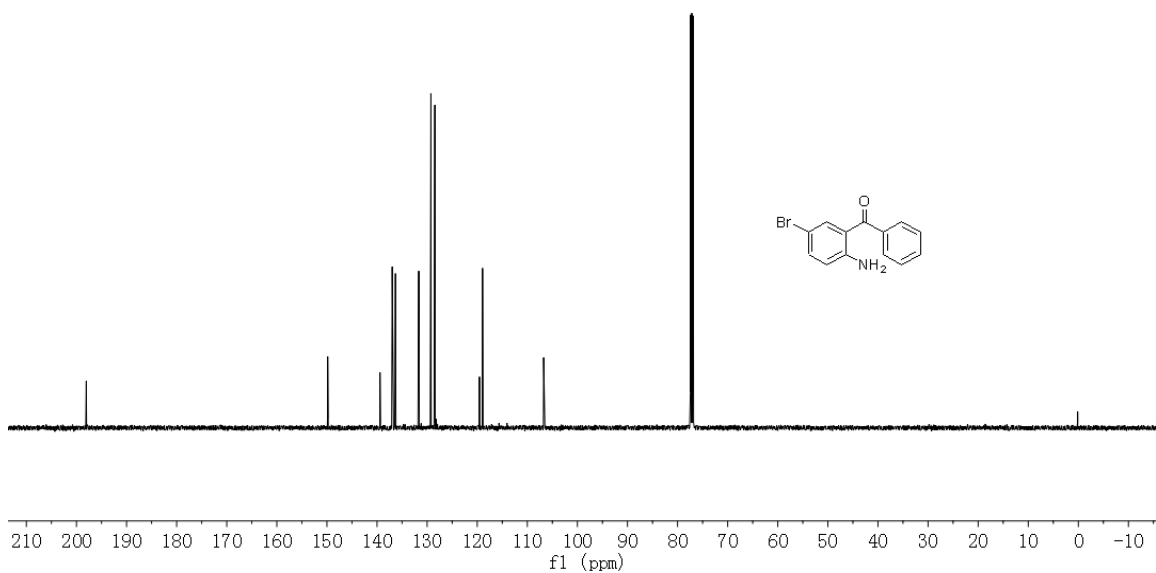

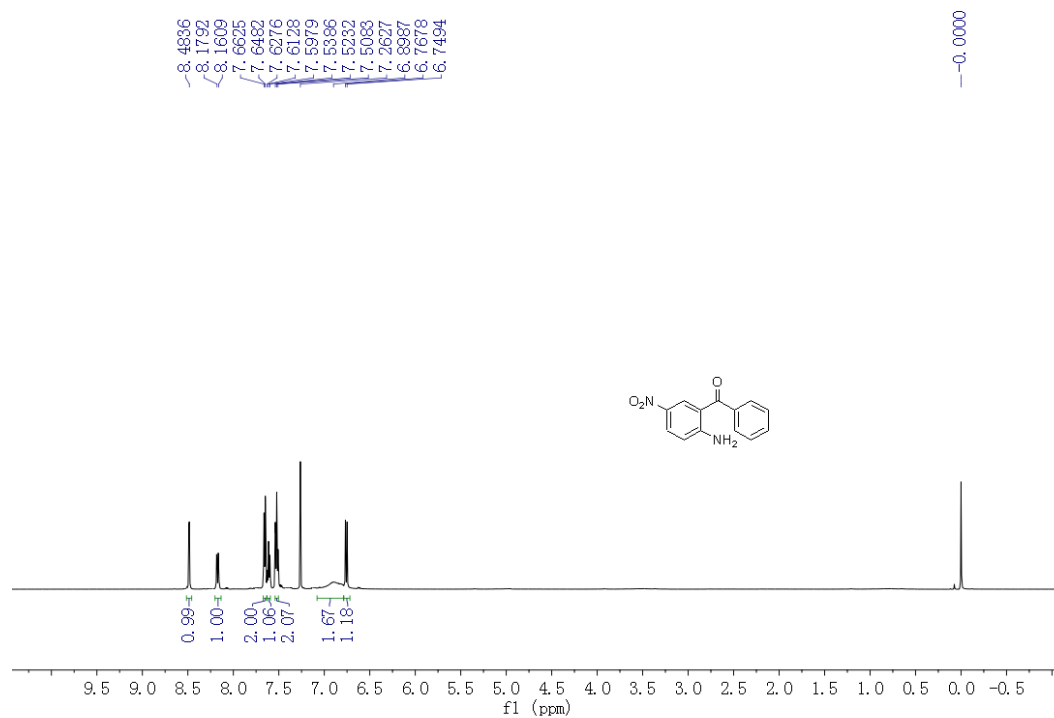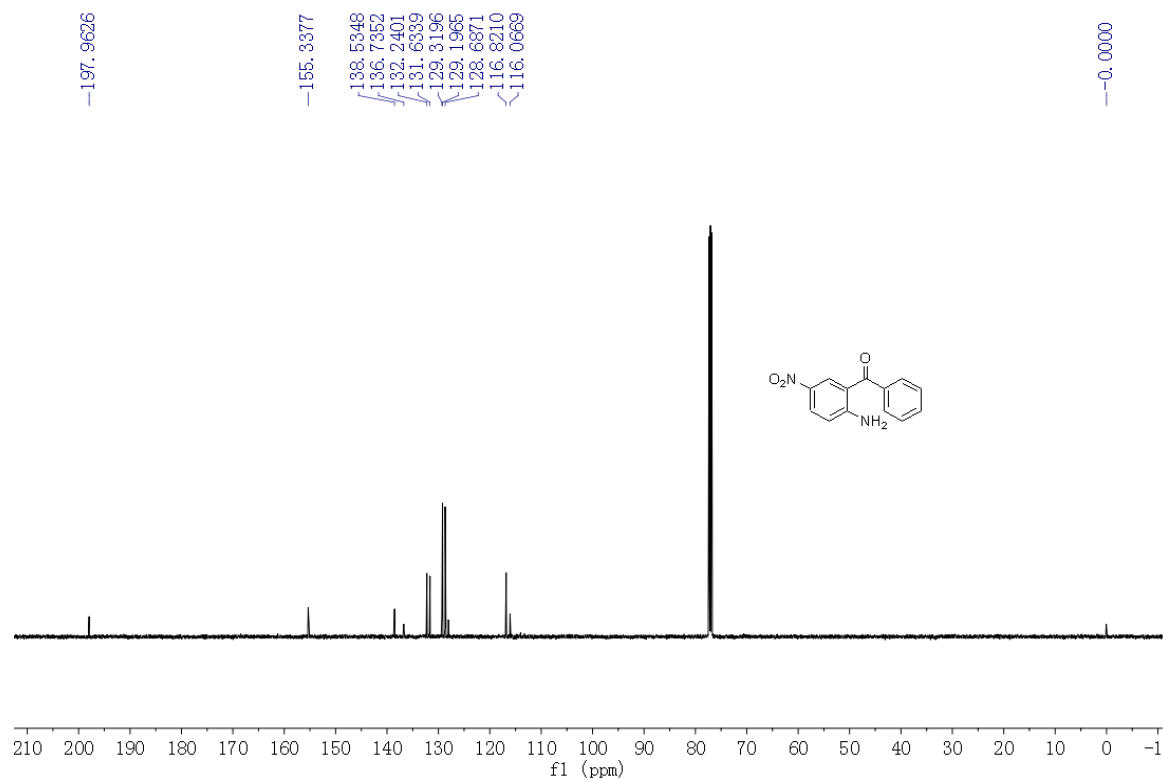

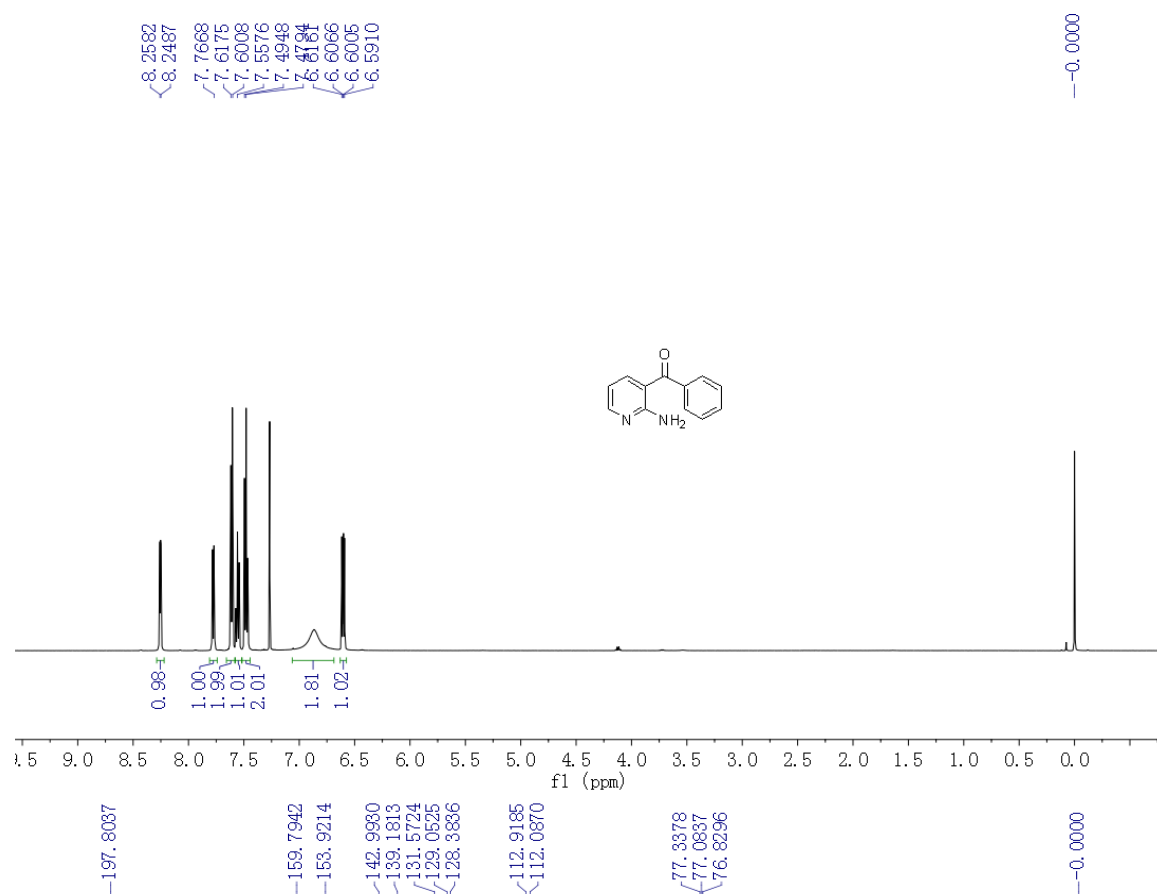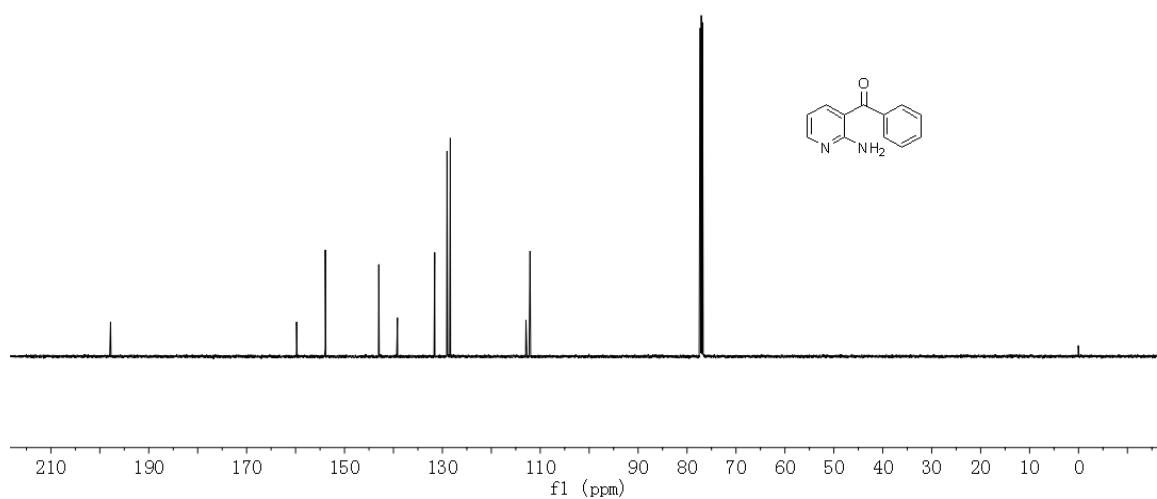

Supplement: Supplementary file 1 [file molecules-19-06439-s001.pdf]
